# Multimorbidity, polypharmacy and inappropriate prescribing in elderly patients with atrial fibrillation: A report from the China Atrial Fibrillation Registry Study

**DOI:** 10.3389/fcvm.2022.988799

**Published:** 2022-09-06

**Authors:** Xueyuan Guo, Mengmeng Li, Xin Du, Chenxi Jiang, Songnan Li, Ribo Tang, Caihua Sang, Ronghui Yu, Deyong Long, Jianzeng Dong, Changsheng Ma

**Affiliations:** ^1^Department of Cardiology, Beijing Anzhen Hospital, Capital Medical University, National Clinical Research Center for Cardiovascular Diseases, Beijing, China; ^2^Department of Cardiology, Peking University Third Hospital, Beijing, China

**Keywords:** atrial fibrillation, elderly patients, multimorbidity, polypharmacy, inappropriate prescribing

## Abstract

**Background:**

Multimorbidity, polypharmacy and inappropriate prescribing is common in elderly patients worldwide. We aimed to explore the current status of multimorbidity, polypharmacy and the appropriateness of pharmacological therapy among elderly patients with atrial fibrillation (AF) in China.

**Materials and methods:**

We randomly selected 500 patients aged 65 years or older from the China AF Registry study. Multimorbidity was defined as ≥2 comorbidities and polypharmacy was defined as ≥5 long-term prescribed drugs. Appropriateness of prescribing was evaluated using the Screening Tool of Older People’s Prescriptions/Screening Tool to Alert to Right Treatment (STOPP/START) criteria version 2. Patients’ attitudes toward polypharmacy were evaluated by the Patients’ Attitudes Towards Deprescribing (PATD) questionnaire.

**Results:**

Among the 500 patients included (mean age 75.2 ± 6.7 years, 49.0% male), 98.0% had multimorbidity and 49.4% had polypharmacy. The prevalence of potentially inappropriate medications (PIMs) and potential prescribing omissions (PPOs) was 43.6% (*n* = 218) and 71.6% (*n* = 358), respectively. Traditional Chinese medicine attributed largely to PIMs. Anticoagulants were the most common PPOs. Many clinical factors increased the risk of PIMs and PPOs. However, polypharmacy increased the risk of PIMs (OR 2.70, 95%CI 1.78–4.11; *p* < 0.0001), but not PPOs. In addition, 73.7% patients with polypharmacy were willing to have one or more of their medications prescribed if advised by their doctor.

**Conclusion:**

Multimorbidity and polypharmacy were highly prevalent in elderly patients with AF in China. A high prevalence of inappropriate prescribing was also observed. Therefore, much more attention should be paid to the serious health problem in the elderly population.

## Introduction

As a popular cardiovascular disease, atrial fibrillation (AF) poses significant burden to our society, and about five million new cases are diagnosed annually (1, 2). As is well known, the prevalence and incidence of AF increases significantly with age (3). Older adults often have multiple clinically significant comorbidities, for example, hypertension, hyperlipidemia, diabetes mellitus and coronary artery disease, with the need for multiple medications for symptoms and diseases control (4, 5). Therefore, polypharmacy, generally defined as administration of five or more concomitant drugs, is common in elderly patients with AF (6, 7). However, polypharmacy is associated with increased risk of inappropriate medication use, adverse drug reactions, drug-drug interactions, and poor clinical outcomes (8–10). In fact, patients’ attitudes toward their medications exert a great impact on optimizing therapy (8).

Currently, data is limited as to the current status of polypharmacy and the appropriateness of pharmacological therapy among Asian patients with AF. In this study, we aimed to (1) describe the patterns of comorbidities and medications among elderly patients with AF; (2) identify potentially inappropriate medications (PIMs), potential prescribing omissions (PPOs) and the associated factors in this population; and (3) measure patients’ attitudes toward prescribing.

## Materials and methods

### Study design and participants

This was a cross-sectional survey of 500 randomly selected participants of the China AF Registry (China-AF) study enrolled between 2011 and 2017. The rationale and design of the study have been previously published (11). In brief, it is an on-going, prospective, hospital-based registry study of AF patients in Beijing, China. Out-patients and in-patients from 19 tertiary and 12 non-tertiary hospitals were enrolled. All the enrolled patients had AF documented via either ECG or Holter within the past 6 months. Patients with transient and reversible AF, life expectancy less than 1 year, and those diagnosed with rheumatic heart disease were excluded from the study. All participants were managed by their local physicians or general practitioners during follow up.

Participants were eligible for inclusion in this survey if they were aged 65 years or older, and did not have moderate or severe cognitive impairment. We randomly selected eligible individuals from the pool of patients registered in the China-AF study using a computer-generated random number method.

The China-AF study was reviewed and approved by the Ethics committee of Beijing Anzhen Hospital. All patients provided their written informed consent to be followed up and contacted for future sub-studies. We obtained a separate ethic approval from the same hospital for this study. Randomly selected participants were contacted and provided additional verbal consent to be involved in this sub-study for further information collection.

### Data collection and measurement

Key data elements like demographic and socioeconomic characteristics, medical conditions and medications related to AF were collected by trained cardiologists and research coordinators from medical records and interviews with patients at the time of enrollment, and updated every 6 months. Further information of general comorbidities and medication use for this analysis were collected via participant self-report during sampling investigation. Recorded medications included all over-the-counter, prescription and herbal/complementary medicines with long-term use (≥1 month). Similar to previous studies (6, 12), we defined polypharmacy as ≥5 long-term prescribed drugs and multimorbidity as 2 or more long-term health conditions (≥3 months).

Inappropriate prescribing encompasses PIMs and PPOs. We used the Screening Tool of Older People’s Prescriptions/Screening Tool to Alert to Right Treatment (STOPP/START) criteria version 2 to evaluate the appropriateness of prescribing (13, 14). The criteria, including 80 STOPP items and 34 START items, are widely used to screen PIMs and PPOs respectively (13). And the screening tool has been translated and adapted from English into several languages to facilitate the application worldwide (15, 16).

For our study population and setting, we made two modifications to the STOPP/START tool. Firstly, we added a condition for START criteria A1: Vitamin K antagonists or direct thrombin inhibitors or factor Xa inhibitors in the presence of chronic AF with high-risk of stroke. High risk patients were defined by a CHA2DS2-VASc score (one point each for hypertension, heart failure, diabetes mellitus, vascular disease, age between 65 and 74 years, female sex; two points each for prior stroke/transient ischemic attack/thromboembolism and age ≥75 years) ≥2 in male and ≥3 in female (17). Secondly, because of insufficient evidence of traditional Chinese medicine (TCM) in the management of AF, we defined the use of TCM for AF as PIMs, referred to the STOPP criteria “drugs without an evidence-based clinical indication” (18).

We used the Patients’ Attitudes Towards Deprescribing (PATD) questionnaire to measure patients’ attitudes and beliefs toward their medicines and potential deprescribing (19). And “deprescribing” is referred as appropriate cessation or reduction of medications to optimize medication regimens (20). In consideration of different acceptance of medication and surrounding beliefs between polypharmacy and non-polypharmacy individuals, the PATD questionnaire was administrated only in participants who were currently taking more than five medications. The questionnaire was originally developed and validated in Australia. In our study, it was translated into the Chinese (with permission from PATD authors) by three bilingual investigators. The translated version was then piloted in 50 older adults to ensure that the wording and content was appropriate for the population and setting.

### Statistical analysis

Categorical variables were reported as frequencies and percentages. And continuous variables were expressed as mean ± standard deviation or medians with interquartile range (IQR), as appropriate. To observe the patterns of polypharmacy and PIMs/PPOs, patients were stratified by age (65–69 years, 70–74 years, 75–79 years, and ≥80 years) and number of comorbidities. The trend was tested using trend Chi-square tests.

Multivariable logistic regression was performed to identify independent risk factors associated with PIMs/PPOs. Apart from variables that were significant in the univariate analysis, we also adjusted for variables with potential influence, regardless of their statistical significance at univariate analysis. Potential confounders included age, sex, hospital level, AF type, education, health insurance coverage, cardiovascular disease, endocrine disease, previous bleeding and systemic embolism. For the first 10 questions of PATD questionnaire, participants who “agreed” or “strongly agreed” were grouped together and analyzed against those responding “unsure,” “disagree” or “strongly disagree.” All data was processed using SPSS (version 21.0; SPSS Inc., Chicago, IL, United States. *P*-value < 0.05 was considered statistically significant.

## Results

### Participant characteristics

Characteristics of randomly selected participants were shown in Table 1. The mean age was (75.2 ± 6.7) years, and 49.0% of the participants were men. Among the participants, 249 (49.8%) had persistent AF and 353 (70.6%) were high school or college educated. The vast majority of participants (96.8%) were totally or partially covered by medical insurance.

**TABLE 1:** Demographic and clinical characteristics of enrolled patients (*N* = 500).

Characteristics	n (%)
Age (years)*	75.2 ± 6.7
Male	245 (49.0)
**Education**	
Did not complete high school	147 (29.4)
Completed high school	228 (45.6)
College educated	125 (25.0)
**Health insurance coverage**	
Total	47 (9.4)
Partially	437 (87.4)
None	16 (3.2)
**Hospital level**	
Tertiary	363 (72.6)
Non-tertiary	137 (27.4)
**Type of atrial fibrillation**	
Paroxysmal atrial fibrillation	251 (50.2)
Persistent atrial fibrillation	249 (49.8)

*Mean ± standard deviation.

### Burden of comorbidities

**FIGURE 1:**
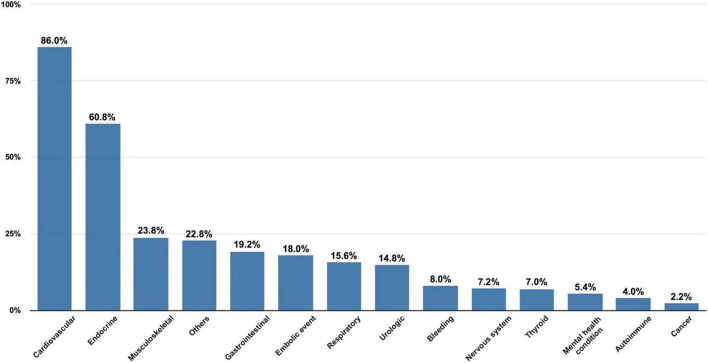
Prevalence of comorbidities in participants. Autoimmune: systemic lupus erythematosus, Sjögren’s syndrome, psoriatic arthritis, rheumatoid arthritis and Takayasu’s arteritis; Bleeding: a history of bleeding with clinical symptoms significantly; Cancer: all kinds of malignant tumors; Cardiovascular: atherosclerosis, arrhythmia other than atrial fibrillation, coronary artery disease, congestive heart failure, hypertension, cardiomyopathy, peripheral arterial disease, deep vein thrombosis; Endocrine: diabetes mellitus, hyperlipidemia, hyperuricemia, hyperhomocysteinemia, gout and adrenal disease; Gastrointestinal: chronic gastritis, ulcer disease, gastroesophageal reflux disease, Crohn’s disease, ulcerative colitis, hepatobiliary calculus, liver dysfunction and cirrhosis; Musculoskeletal: spondyloarthritis, fibromyalgia, osteoarthritis, osteoporosis and osteopenia; Nervous system: dementia, Parkinson’s disease, Alzheimer’s disease, epilepsy, vertigo, migraine and peripheral neuropathy; Others: anemia, implantation of cardiac implantable electronic devices, chronic pain, chronic infectious disease and gynecological disease; Mental health condition: depression, anxiety, phobias, bipolar disorder and sleep disorder; Respiratory: asthma, chronic bronchitis, emphysema, chronic obstructive pulmonary disease chronic rhinitis, allergic rhinitis, obstructive sleep apnea and pulmonary embolism; Embolic event: a history of stroke, transient ischemic attack or systemic embolism; Thyroid: hyperthyroidism and hypothyroidism; Urologic: chronic kidney disease, urolithiasis and benign prostatic hyperplasia.

Cardiovascular conditions accounted for the largest burden of comorbidities (86.0%), followed by endocrine conditions (60.8%) and musculoskeletal conditions (23.8%) (Figure 1). The most common cardiometabolic comorbidities were hypertension (74.8%), hyperlipidemia (43.0%), diabetes mellitus (28.8%), coronary artery disease (27.8%), congestive heart failure (17.6%), and stroke/transient ischemic attack/peripheral embolism (18.0%). Almost all participants (98.0%) had at least one comorbidity in addition to AF, 32.6% had five or more medical conditions and 1.4% had 10 or more. The median number of comorbidities was 4 (IQR 2–5).

### Polypharmacy

**FIGURE 2:**
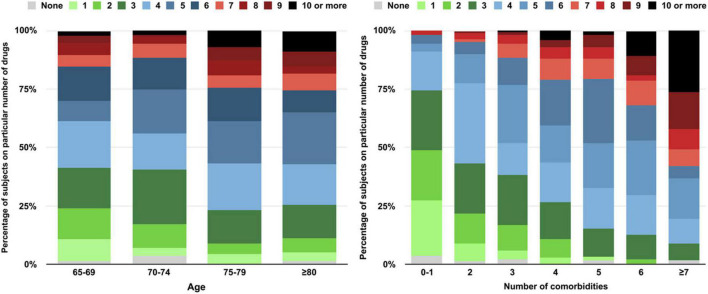
Number of medications taken by participants stratified by age and number of comorbidities.

The median number of concomitant medication types and tablets/capsules taken daily was 4 (IQR, 3–6) and 8 (IQR, 4–13). About half of the patients (49.4%) received five or more different types of medications (polypharmacy). The number of medications increased significantly with age (*p* < 0.05) and comorbidities (p < 0.001). Polypharmacy was present in 38.8, 44.1, 56.7, and 57% of participants aged 65–69, 70–74, 75–79 years, and aged ≥80 years respectively, and from 9.1 to 48.0%, 66.1 and 80.7% in patients with 0–1, 3, 5, and ≥7 comorbidities (Figure 2).

The most common used drugs were β-blockers (55.4%), followed by statins (47.8%) and angiotensin-converting enzyme inhibitors/angiotensin receptor antagonists (ACEIs/ARBs, 37.8%). Oral anticoagulant agents were used in 41.6% of participants (32.2% on warfarin and 9.4% on non-vitamin K antagonist oral anticoagulants). More than a third of participants were taking TCM which was prescribed for symptom relief or an alternative for anticoagulants (Table 2).

**TABLE 2:** Distribution of most commonly used drugs of enrolled patients (*N* = 500).

Drug class	n (%)
β-blockers	277 (55.4)
Statins	239 (47.8)
Traditional Chinese medicine	193 (38.6)
ACEIs/ARBs	189 (37.8)
Aspirin	186 (37.2)
Warfarin	161 (32.2)
Calcium channel blockers	129 (25.8)
Hypoglycemic drugs	98 (19.6)
Nitrates	98 (19.6)
Diuretics	63 (12.6)
Class IIb/IIIa antiarrhythmic drugs	61 (12.2)
Digoxin	52 (10.4)
NOACs	47 (9.4)
Antiplatelet agents other than aspirin	45 (9.0)

ACEIs/ARBs, angiotensin-converting enzyme inhibitors/angiotensin receptor antagonists; NOACs, non-vitamin K antagonist oral anticoagulants.

### Inappropriate prescribing (potentially inappropriate medications and potential prescribing omissions)

According to the STOPP criteria, a total of 256 PIMs events were identified, and PIMs use occurred in 43.6% of participants. Moreover, 38.4, 2.8, and 2.4% were prescribed one, two, and three PIMs, respectively. No patient was prescribed four or more PIMs (Table 3). The most frequent PIM used was TCM, and the rate was as high as 38.6% in all enrolled patients, which also account for 75.3% of all PIMs events (Supplementary Table 1).

**TABLE 3:** Distribution of PIMs between different age groups according to the STOPP criteria.

Age group	Patients number	Median number of medications (IQR)	Total PIMs events, n	≥1 PIMs *n* (%)	1 PIM n (%)	2 PIMs n (%)	3 PIMs n (%)	≥ 4 PIMs n (%)
65–69	129	4 (3–6)	54	48 (37.2)	43 (33.3)	4 (3.1)	1 (0.8)	0 (0.0)
70–74	111	4 (3–6)	52	50 (45.0)	48 (43.2)	2 (1.8)	0 (0.0)	0 (0.0)
75–79	111	5 (4–6)	66	54 (48.6)	46 (41.4)	4 (3.6)	4 (3.6)	0 (0.0)
≥80	149	5 (3–7)	84	66 (44.3)	55 (36.9)	4 (2.7)	7 (4.7)	0 (0.0)
Total	500	4 (3–6)	256	218 (43.6)	192 (38.4)	14 (2.8)	12 (2.4)	0 (0.0)

IQR, interquartile range; PIMs, potentially inappropriate medications; STOOP, screening tool of older people’s prescriptions.

According to the START criteria, a total of 637 PPOs events were identified, and the prevalence of PPOs was 71.6%. One, two, three, and four or more PPOs were identified in 35.2, 22.2, 9.6, and 4.6% of participants, respectively (Table 4). Over half (58.1%) of high-risk participants did not receive adequate anticoagulant therapy. Of the 197 participants with systolic heart failure, 124 (62.9%) were not taking ACEIs/ARBs. Similarly, statins were not prescribed in 69 out of 189 (36.5%) participants with an indication for lipid-lowering therapy. And β-blockers were also underused (Supplementary Table 2).

**TABLE 4:** Distribution of PPOs between different age groups according to the START criteria.

Age group	Patients number	Median number of medications (IQR)	Total PPOs events, n	≥1 PPOs n (%)	1 PPO n (%)	2 PPOs n (%)	3 PPOs n (%)	≥4 PPOs n (%)
65–69	129	4 (3–6)	110	75 (58.1)	48 (37.2)	21 (16.3)	4 (3.1)	2 (1.5)
70–74	111	4 (3–6)	135	75 (67.5)	39 (35.1)	17 (15.3)	14 (12.6)	5 (4.5)
75–79	111	5 (4–6)	151	91 (82.0)	46 (41.4)	32 (28.8)	11 (10.0)	2 (1.8)
≥80	149	5 (3–7)	241	117 (78.5)	43 (28.9)	41 (27.5)	19 (12.7)	14 (9.4)
Total	500	4 (3–6)	637	358 (71.6)	176 (35.2)	111 (22.2)	48 (9.6)	23 (4.6)

IQR, interquartile range; START, screening tool to alert to right treatment; PPOs, potential prescribing omissions.

### Factors associated with potentially inappropriate medications and potential prescribing omissions

After adjustment for potential confounders, the following factors were found to be independently associated with PIMs: education level [college education: odds ratio (OR) 2.11, 95% confidence interval (CI) 1.16–3.85; *p* = 0.04], paroxysmal AF (OR 1.58, 95%CI 1.02–2.46; *p* = 0.04), gastrointestinal disease (OR 1.78, 95%CI 1.09–2.92; *p* = 0.02), mental health condition (OR 4.38, 95%CI 1.54–12.50; *p* < 0.001), and polypharmacy (OR 2.70, 95%CI 1.78–4.11; *p* < 0.001) (Table 5).

**TABLE 5:** Multivariable analysis of clinical factors associated with PIMs and PPOs.

	PIMs			PPOs		
Factors	All patients n/N (%)	Adjusted OR (95%CI)	*P-Value*	All patients n/N (%)	Adjusted OR (95%CI)	*P-Value*
**Age**						
≥75	120/260 (46.2)	1.17 (0.77–1.78)	0.47	208/260 (80.0)	1.68 (1.04–2.70)	0.03
65–74	98/240 (40.8)	1.00		150/240 (62.5)	1.00	
**Gender**						
Female	114/255 (44.7)	1.05 (0.70–1.59)	0.81	180/255 (70.6)	0.80 (0.50–1.30)	0.37
Male	104/245 (42.4)	1.00		178/245 (72.7)	1.00	
**Hospital level**						
Non-tertiary	53/137 (38.7)	0.84 (0.50–1.42)	0.52	123/137 (89.8)	5.55 (2.74–11.24)	<0.001
Tertiary	165/363 (45.5)	1.00		235/363 (64.7)	1.00	
**Health insurance coverage**						
Total	24/47 (51.1)	1.19 (0.58–2.43)	0.89	34/47 (72.3)	1.04 (0.23–4.70)	0.97
Partially	189/437 (43.2)	1.11 (0.29–4.30)		313/437 (71.6)	0.94 (0.26–3.39)	
None	5/16 (31.3)	1.00		11/16 (68.8)	1.00	
**Education**						
College educated	67/125 (53.6)	2.11 (1.16–3.85)	0.04	86/125 (68.8)	0.84 (0.45–1.56)	0.70
High school educated	100/228 (43.9)	1.58 (0.95–2.63)		154/228 (67.5)	0.74 (0.36–1.50)	
Under high school	51/147 (34.7)	1.00		118/147 (80.3)	1.00	
**AF type**						
Paroxysmal	122/251 (48.6)	1.58 (1.02–2.46)	0.04	179/251 (71.9)	2.26 (1.35–3.76)	0.001
Persistent	96/249 (38.6)	1.00		179/249 (71.3)	1.00	
Catheter ablation*						
Yes	52/99 (52.5)	1.45 (0.86–2.44)	0.16	67/99 (67.7)	–	–
No	166/401 (41.4)	1.00		291/401 (72.6)	–	
**Cardiovascular disease**						
Yes	190/430 (44.2)	1.17 (0.63–2.20)	0.62	331/430 (77.0)	4.90 (2.55–9.42)	<0.001
No	28/70 (40.0)	1.00		27/70 (38.6)	1.00	
**Endocrine disease**						
Yes	138/304 (45.4)	1.10 (0.72–1.70)	0.66	217/304 (71.4)	0.77 (0.46–1.29)	0.32
No	80/196 (40.8)	1.00		141/196 (71.9)	1.00	
**Previous bleeding**						
Yes	18/40 (45.0)	1.09 (0.54–2.23)	0.81	34/40 (85.0)	3.23 (1.14–9.20)	0.03
No	200/460 (43.5)	1.00		324/460 (70.4)	1.00	
**Previous systematic embolism**						
Yes	35/90 (38.9)	0.64 (0.37–1.08)	0.09	75/90 (83.3)	1.60 (0.82–3.10)	0.17
No	183/410 (44.6)	1.00		283/410 (69.0)	1.00	
**Respiratory disease ^†^**						
Yes	36/78 (46.2)	–	–	64/78 (82.1)	1.96 (0.97–3.96)	0.06
No	182/422 (43.1)	–		294/422 (69.7)	1.00	
Gastrointestinal disease*						
Yes	56/96 (58.3)	1.78 (1.09–2.92)	0.02	71/96 (74.0)	–	-
No	162/404 (40.1)	1.00		287/404 (71.0)	–	
**Urologic disease**						
Yes	41/74 (55.4)	1.58 (0.90–2.76)	0.11	64/74 (86.5)	1.83 (0.84–3.99)	0.13
No	177/426 (41.5)	1.00		294/426 (69.0)	1.00	
**Thyroid disease^†^**						
Yes	17/35 (48.6)	–	–	19/35 (54.3)	0.47 (0.21–1.04)	0.06
No	201/465 (43.2)	–		339/465 (72.9)	1.00	
Psychological disease*						
Yes	22/27 (81.5)	4.38 (1.54–12.50)	<0.001	22/27 (81.5)	–	–
No	196/473 (41.4)	1.00		336/473 (71.0)	-	
**Polypharmacy**						
Yes	138/247 (55.9)	2.70 (1.78–4.11)	<0.001	186/247 (75.3)	1.03 (0.63–1.68)	0.90
No	80/253 (31.6)	1.00		172/253 (68.0)	1.00	
**Multimorbidity**						
≥2	201/445 (45.2)	1.19 (0.56–2.54)	0.66	332/445 (74.6)	1.88 (0.85–4.18)	0.12
0–1	17/55 (30.9)	1.00		26/55 (47.3)	1.00	

AF, atrial fibrillation; PIMs, potentially inappropriate medications; PPOs, potential prescribing omissions. *The factor was not taken into the multivariable analysis of PPOs. ^†^The factor was not taken into the multivariable analysis of PIMs.

Independent predictors of PPOs were age over 75 years (OR 1.68, 95%CI 1.04–2.70; *p* = 0.03), non-tertiary hospital management (OR 5.55, 95%CI 2.74–11.24, *p* < 0.001), paroxysmal AF (OR 2.26, 95%CI 1.35–3.76, *p* = 0.001), history of cardiovascular diseases (OR 4.90, 95%CI 2.55–9.42; *p* < 0.0001), and history of bleeding (OR 3.23, 95%CI 1.14–9.20; *p* = 0.03). No significant association was observed between polypharmacy and PPOs (OR 1.03, 95%CI 0.63–1.68; *p* = 0.90) (Table 5).

### Attitudes toward deprescribing

**FIGURE 3:**
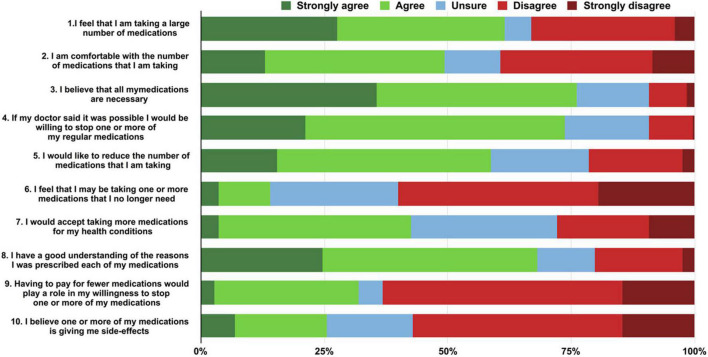
Participants responses to the PATD (patients’ attitudes toward deprescribing) questionnaire (questions 1–10).

The PATD questionnaire was administrated to 247 participants with polypharmacy, and the response was presented in Figure 3. 61.0% of participants agreed that they were taking a large number of medications (Question 1) and 50.6% reported to be uncomfortable with current number of medications (Question 2). Approximately three quarters (73.7%) of participants agreed or strongly agreed that they would be willing to have one or more of their medications prescribed if advised by their doctor (Question 4). Experiencing a side-effect and financial burden as a consideration for deprescribing were reported by 25.5 and 32.0% of participants (Question 9 and 10). At the meantime, most participants (76.1%) believed the medications they were taking were necessary (Question 3) and 42.5% were willing to accept taking more medications for their health conditions (Question 7). The maximum ideal number of medications was reported in 30.8% for 8 tablets/capsules taken daily, 25.6% for 12 and 21.1% for 16 (Question 13) (Supplementary Table 3).

## Discussion

In this study, we found that multimorbidity was present in almost all older adults with AF. Nearly half of the participants had polypharmacy and both over- and under-treatment were identified in this population. More than half patients did not receive proper anticoagulant therapy. ACEIs/ARBs, β-blockers, and statins were also underused according to the STOPP/START criteria. However, agents without sufficient clinical indications were commonly used. About three quarters of participants with polypharmacy expressed the willingness to stop one or more medications under the guidance of the clinician.

We confirmed multimorbidity was common in elderly AF patients, and the rate was as high as 98.0%. Indeed, multimorbidity prevalence in AF was ranged from 69.5 to 98%, for different study setting and population (21–23). A Swedish study of 272186 AF patients reported 69.5% prevalence of at least one other comorbidity (21). A study in Belgium reported that 92% of patients with AF had 3 or more comorbidities (22). A study in the United States using the data from National Health and Wellness survey (NHWS) found that 98% of participants with AF had at least one additional comorbidity (23). Generally, the number of comorbidities increases with age in the older population (24). And in our study the mean age of the AF patients was 75.2 years old.

Moreover, we identified the general comorbidity pattern and drew a relatively comprehensive picture of the total disease burden in the elderly AF patients in China, which would help to improve treatment and health outcomes. The incidence of cardiovascular diseases other than AF was 86.0%, which exerted the biggest comorbidity burden in our population, and hypertension was the most prevalent comorbidity. In fact, hypertension is closely associated with the development of AF (25). Unlike with studies from western countries (23, 26), we found that the incidence of non-cardiometabolic conditions was low in our study.

Polypharmacy is becoming increasingly common in clinical practice due to the aging society and the high burden of comorbidities (27, 28). Indeed, multimorbidity acts as a driver of polypharmacy. In patients with high morbidity, the proportion of polypharmacy is also high (29). The prevalence of polypharmacy was 49.4% in our study. Previous studies have reported rates of polypharmacy in 40–77% of patients with AF, with varying prescription patterns and inclusion and exclusion criteria (6, 12, 30). The potential harms of polypharmacy have been widely reported, including inappropriate prescribing, increased risk of adverse drug events and poor clinical outcomes (8–10, 31). Unfortunately, polypharmacy may bring unique risk in older adults with AF. Several studies found that polypharmacy was an independent risk factor for bleeding and thromboembolic events in patients with AF (6, 12).

Inappropriate prescribing, including PIMs and PPOs, was also prevalent in our study population. Almost half of our enrolled participants were taking at least one PIMs, such as TCM. In fact, some TCMs were reported to have antithrombotic effect and interact with anticoagulant agents, for example, danshen (Salvia miltiorrhiza), danshen (Salvia miltiorrhiza), Asian ginseng (Panax ginseng) and so on (18, 32). Concomitant use of TCM would increase the risk of bleeding. Thus, clinicians may need to specifically ask about TCM use before prescribing anticoagulants.

Currently, anticoagulant therapy is recommended among AF patients with high or moderate risk of stroke (17). In this study, 90.8% of participants had an indication for anticoagulant therapy, but only 41.9% were prescribed these medications (35.5% on warfarin and 6.4% on NOACs). This proportion was similar to the number we observed in our previous China-AF sub-study (33). Although great improvement in anticoagulant usage has been achieved in recent years, the gap is still large between clinical practice and guideline recommended therapy in China (33, 34). As stated in our previous study (33), There are some reasons for the underuse of anticoagulants. First, the high risk of bleeding restricts use of anticoagulants in the elderly. Second, patient education and monitoring are inadequate, which impairs the clinicians from prescribing anticoagulants and patients to adhere to therapy. Compared with warfarin, the rate of NOACs was much lower, and the high cost of NOACs may be the reason, because NOACs are unaffordable for the vast majority of patients in China. Meanwhile, ACEIs/ARBs, β-blockers and statins were the most common PPOs in this study, which was consistent with prior data (34). It indicated that the importance of cardiovascular risk factor modification is often neglected. Thus, integrated care is in urgent need in the management of AF patients.

We identified that polypharmacy as well as several medical conditions, such as gastrointestinal disease and mental health condition, increased the risk of PIMs. This may explain the common overprescribing of drugs in specific conditions, such as long-term benzodiazepine use. On the other hand, it also indicated that increased complexity of the disease can lead to inappropriate prescribing (35). Furthermore, PIMs were more common in patients with high education level. The possible reason may be that patients with high education level always have good economic conditions, and they prefer to take TCM as complementary and alternative medicine. In addition, we observed that polypharmacy could not decrease the risk of PPOs. By reviewing the most commonly prescribed PIMs/PPOs, we assumed that the occurrence of PPOs (including anticoagulants, ACEIs/ARBs, etc.) might result in worse clinical outcomes than PIMs (like TCM) in older AF patients. Therefore, an evidence-based drug therapy but within limited number of concomitant medications is vital for this population. Single-pill combination may be a good solution for all the questions.

Inappropriate medication use in older patients is common, which indicates deprescribing is not happening as often as it should. Approximately three quarters of our participants with polypharmacy were willing to have one or more of their medications prescribed if their doctor said it was possible. In fact, deprescribing involves an implicit partnership between the doctor and the patient (36). Not only in China, older individuals in the other countries are also eager to undertake deprescribing, especially when they have a large number of medications, experience side effects or feel some medications are unnecessary (36–38). A meta-analysis of 40 studies and 10,816 participants found that the proportion of patients who agreed or strongly agreed with deprescribing was as high as 84% (39).

There are many factors influencing the patients’ attitudes toward medications, such as insurance coverage status and physician trust (40). Our data suggested that patients’ attitudes and beliefs were important factors in prescribing and deprescribing. Given the complicated nature and associated risks of polypharmacy, it is important to optimize the medication regimen in older adults with AF. In particular, this is a population with high prevalence of cognitive decline (41), which can impact self-medication management. Good communication about the reasons for prescribing and/or deprescribing may help facilitate shared-decision making with older adults and their caregivers. Compared with taking multiple pills, polypill-based regimens have emerged as a promising strategy to increase patient adherence and reduce burden (42). New technologies such as mobile applications may also facilitate optimizing medications (43).

## Limitations

There are some limitations of our study. First, the sample population was recruited from hospitals in Beijing, and the majority of them were from high-volume tertiary medical centers. This may produce biased participant selection, for patients who attend tertiary hospitals usually have a high social economic status and degree of education. Second, our data was patient self-reported, and the estimate of polypharmacy was not validated. Third, the STOPP/START criteria version 2 was published in 2014, and due to the expanding therapeutics evidence base, it could not include every detail on potentially inappropriate prescribing. Finally, the PATD questionnaire was originally designed and validated in Australia, while we conducted piloting in our population, we did not conduct a formal validation process.

## Conclusion

Multimorbidity and polypharmacy were highly prevalent in elderly patients with AF in China. Inappropriate prescribing, including over- and under-treatment, was common in this population. Moreover, many patients expressed their willingness to deprescribing. Thus, drug regimens should be adjusted timely, to satisfy the need of the patients. In the future, much more attention should be paid to the elderly AF population.

## Data availability statement

The raw data supporting the conclusions of this article will be made available by the authors, without undue reservation.

## Ethics statement

The studies involving human participants were reviewed and approved by the Ethics committee of Beijing Anzhen Hospital. The patients/participants provided their written informed consent to participate in this study. Written informed consent was obtained from the individual(s) for the publication of any potentially identifiable images or data included in this article.

## Author contributions

All authors listed have made a substantial, direct, and intellectual contribution to the work, and approved it for publication.
