# Supplementary material for: Multimorbidity, polypharmacy and inappropriate prescribing in elderly patients with atrial fibrillation: A report from the China Atrial Fibrillation Registry Study
Source: Front Cardiovasc Med. 2022 Sep 6;9:988799. doi: 10.3389/fcvm.2022.988799 (PMC9485537; doi:10.3389/fcvm.2022.988799)
Supplement: Supplementary file 1 [file Data_Sheet_1.docx]

**SUPPLEMENTAL MATERIAL**

**Supplemental Table 1**. Prevalence of the most frequently prescribed PIMs according to the STOPP criteria

| Description | Subgroup *  n/N (%) | Overall  N = 500 (%) |
| --- | --- | --- |
| Any drug prescribed without an evidence-based clinical indication (Traditional Chinese medicine) | 193/500 (38.6) | 38.6 |
| Drugs that predictably increase the risk of falls in elder people | 17/500 (3.4) | 3.4 |
| Benzodiazepines for ≥ 4 weeks | 15/500 (3.0) | 3.0 |
| Aspirin in combination with VKA or NOACs in patients with chronic atrial fibrillation | 8/500 (1.6) | 1.6 |
| Sulphonylureas with a long duration of action with type 2 diabetes mellitus | 5/144 (3.5) | 1.0 |
| Beta-blockers in combination with verapamil or diltiazem | 5/277 (1.8) | 1.0 |

Abbreviations: NOACs, non-vitamin K antagonist oral anticoagulants; PIMs, potentially inappropriate medications; STOPP, screening tool of older people’s prescriptions; VKA, vitamin K antagonist.

* Subgroup indicates patients with a certain condition or specific medications

**Supplemental Table 2**. Prevalence of the most frequent potential prescribing omissions according to the START criteria

| Description | Subgroup*  n/N (%) | Overall N=500 (%) |
| --- | --- | --- |
| VKA or NOACs in the presence of chronic atrial fibrillation | 264/454 (58.1) | 52.8 |
| ACEIs/ARBs with systolic heart failure and/or documented coronary artery disease | 124/197 (62.9) | 24.8 |
| Statin therapy with a documented history of coronary, cerebral or peripheral vascular disease, unless the patient’s status is end-of-life or age is > 85 years. | 69/189 (36.5) | 13.8 |
| Beta-blockers with ischemic heart disease. | 48/139 (34.5) | 9.6 |
| Appropriate beta-blockers with stable systolic heart failure. | 34/88 (38.6) | 6.8 |
| Appropriate antihypertensive therapy | 33/374 (8.8) | 6.6 |
| Alpha-1 receptor blocker and 5-alpha reductase inhibitor with symptomatic prostatism, where prostatectomy is not considered necessary | 23/44 (59.1) | 4.6 |
| Regular inhaled β-2 agonist or antimuscarinic bronchodilator for mild to moderate asthma or COPD | 18/23 (78.3) | 3.6 |
| Vitamin D supplement in patients with known osteoporosis | 15/18 (83.3) | 3.0 |
| Regular inhaled corticosteroid for moderate-severe asthma or COPD, where FEV1 <50% of predicted value and repeated exacerbations requiring treatment with oral corticosteroids | 5/7 (71.4) | 1.0 |

Abbreviations: ACEIs/ARBs, angiotensin-converting enzyme inhibitors/angiotensin receptor antagonists; COPD, chronic obstructive pulmonary disease; NOACs, non-vitamin K antagonist oral anticoagulants; START, screening tool to alert doctors to right treatment; VKA, vitamin K antagonist. * Subgroup indicates patients with a certain condition or specific medications.

**Supplemental Table 3.** Participants responses to the PATD questionnaire (questions 11-15)

| Question | Answer | % |
| --- | --- | --- |
| 11. Have you ever tried to stop a regular medication with the approval of your doctor? | No | 61.1 |
|  | Yes, and I never took it thereafter | 21.1 |
|  | Yes, but I had to start taking it again | 10.5 |
|  | Yes, but I had to take a different medicine | 7.3 |
| 12. How many different tablets or capsules per day would you consider to be a lot? | 5-9 | 12.6 |
|  | 10-14 | 29.1 |
|  | 15-19 | 30.8 |
|  | 20-25 | 15.4 |
|  | > 25 | 12.1 |
| 13. What is the maximum number of tablets or capsules that you would be comfortable taking per day? | 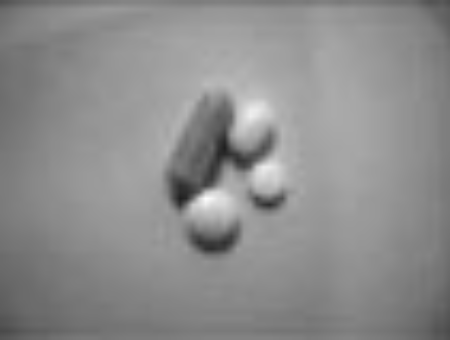(4) | 13.6 |
|  | 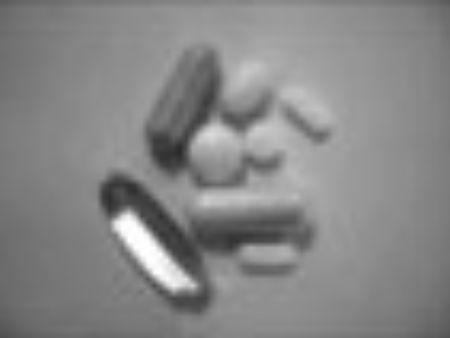(8) | 30.8 |
|  | 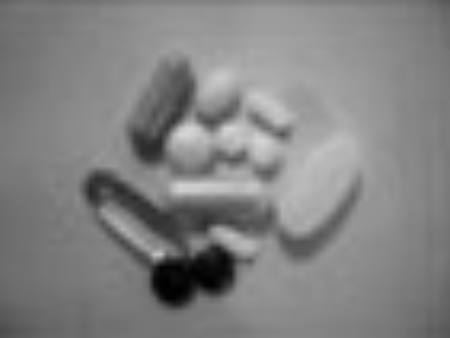(12) | 25.6 |
|  | 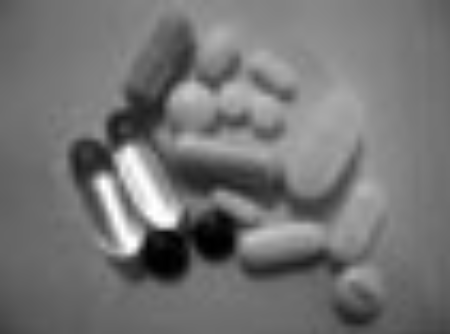(16) | 21.1 |
|  | 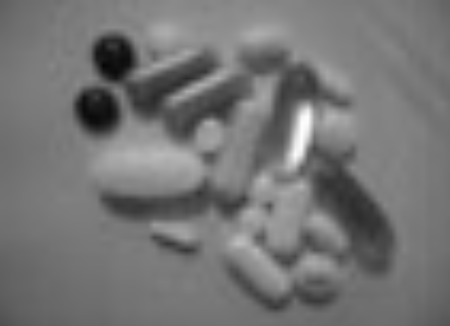(20) | 8.1 |
|  | 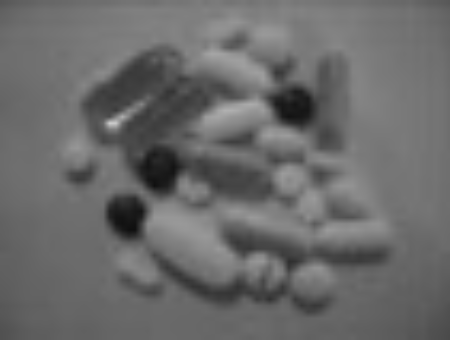(24) | 0.8 |
| 14. If one of your regular medications was stopped, what follow-up would you like? | Face-to-face appointment | 37.2 |
|  | Telephone call | 41.3 |
|  | Contact with a health practitioner when necessary without programmed follow-up | 20.6 |
|  | Written information sent by email | 0.9 |

Abbreviations: PATD, patients’ attitudes towards deprescribing.
